# An ultrasensitive and stretchable strain sensor based on a microcrack structure for motion monitoring

**DOI:** 10.1038/s41378-022-00419-6

**Published:** 2022-09-29

**Authors:** Hao Sun, Xudong Fang, Ziyan Fang, Libo Zhao, Bian Tian, Prateek Verma, Ryutaro Maeda, Zhuangde Jiang

**Affiliations:** 1 State Key Laboratory for Manufacturing Systems Engineering, International Joint Laboratory for Micro/Nano Manufacturing and Measurement Technologies, Collaborative Innovation Center of Suzhou Nano Science and Technology, Xi’an Jiaotong University, Xi’an, 710049 China; 2 School of Mechanical Engineering, Xi’an Jiaotong University, Xi’an, 710049 China; 3 Overseas Expertise Introduction Center for Micro/Nano Manufacturing and Nano Measurement Technologies Discipline Innovation, and Xi’an Jiaotong University (Yantai) Research Institute for Intelligent Sensing Technology and System, Xi’an, China; 4 School of Chemical Engineering, University of Arkansas, Fayetteville, AR 72701 USA

**Keywords:** Sensors, Nanowires

## Abstract

Flexible strain sensors are promising candidates for intelligent wearable devices. Among previous studies, although crack-based sensors have attracted a lot of attention due to their ultrahigh sensitivity, large strain usually causes fractures in the conductive paths. Because of the unstable crack structure, the tradeoff between sensitivity and workable strain range is still a challenge. As carbon nanotubes (CNTs) and silver nanowires (AgNWs) can form a strong interface with the thermoplastic substrate and strengthen the conductive network by capillary force during water evaporation, CNTs and AgNWs were deposited on electrospun TPU fiber mats via vacuum-assisted filtration in this work. The prestretching treatment constructed a microcrack structure that endowed the sensor with the combined characteristics of a wide working range (0~171% strain), ultrahigh sensitivity (a gauge factor of 691 within 0~102% strain, ~2 × 10^4^ within 102~135% strain, and *>*11 × 10^4^ within 135~171% strain), a fast response time (~65 ms), small hysteresis, and superior durability (*>*2000 cycles). Subsequently, the sensing mechanism of the sensor was studied. Distributed microcrack propagation based on the “island-bridge” structure was explained in detail, and its influence on the strain-sensing behavior of the sensor was analyzed. Finally, the sensor was assembled to monitor various vibration signals and human motions, demonstrating its potential applications in the fields of electronic skin and human health monitoring.

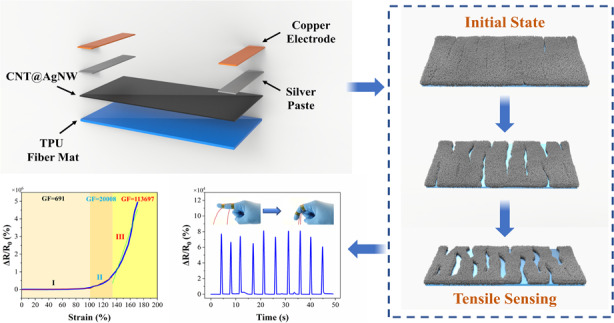

## Introduction

With the rapid development of electronic equipment, wearable devices, such as electronic skin^1,2^, human-computer interaction^3,4^, human health monitoring^5–7^, and robot technology^8,9^, have become a research hotspot in academia and industry. As one of the most widely used sensors in these fields, the piezoresistive flexible strain sensor has attracted a lot of attention due to its simple preparation process and low manufacturing cost^10,11^. To meet practical application requirements, wearable flexible strain sensors should have high sensitivity and a wide working range^12^. Traditional piezoresistive strain sensors made of metal or semiconductors can only stretch and bend in a narrow working range and it is difficult for them to conform to human skin^13,14^. Therefore, polymer substrates with excellent tensile properties have the potential to be the mainstream choice and show exceptional application prospects in the field of flexible electronics^15^.

The preparation of flexible strain sensors is predominantly executed by combining functional conductive materials (CNTs^7,16,17^, graphene^18,19^, AgNWs^20,21^, MXenes^22–24^, etc.) with elastic polymer substrates (such as TPU^25–27^, polydimethylsiloxane (PDMS)^28–30^, and silicone rubber^31–33^). Based on this strategy, many studies have been reported. Yang et al. proposed a stretchable strain sensor based on a Ti_3_C_2_T_x_ MXene nanoparticle-nanosheet hybrid conductive network. Their sensor can monitor 53% strain with a gauge factor (GF) of 178.4 and excellent durability^34^. Li et al. fabricated a flexible strain sensor based on braided graphene ribbon and dragon skin, obtaining good performance by adjusting the cross-region and tilt angle of the ribbon^35^. The sensor showed a superior working range (55.5%) and high sensitivity (GF > 175.16). Although these sensors have excellent sensitivity, their small working range limits the detection of wide-range motion signals. Liu et al. reported a sensor with a wide working range and high linearity^36^. It was obtained by polymer-assisted copper deposition and a novel embroidery method. However, the low sensitivity (GF = 49.5) limited its application in instances of weak signal detection. Through these reported works, the direct combination of functional materials and elastic polymer substrates cannot resolve the conflict between the range and sensitivity of flexible strain sensors. To solve this problem, we urgently need an effective structural design to improve the comprehensive performance of the sensor.

Inspired by crack-shaped slit organs near the joints of spider legs^37,38^, ultrahigh-sensitivity strain sensors based on microcracks have attracted widespread attention for their excellent sensitivity and good stability. Although cracks are generally considered defects to be avoided, they can be effective if they occur in a controlled manner. Through this method, Liu et al.^39^ proposed a simple ultrasound patterning technique to obtain a crack-based strain sensor that avoided transverse Poisson compression through a parallel crack structure. The GF was significantly improved (up to 3.2 × 10^7^). However, the sensor can only test up to 20% strain and be used for precise monitoring of human pulse and throat movement. It is not suitable to detect joint movement. To increase the strain range of the sensor, Li et al. prepared a flexible conductive MXene/cellulose nanocrystal (CNC)-coated TPU nonwoven cloth by simple immersion technology^40^. With adjustable crack density, the sensor showed an 83% sensing range and high sensitivity (GF = 3405). The reported work lacks effective strategies to control crack growth. As proposed by Huang et al.^41^, with increasing strain, the stress concentration at the slit leads to rapid propagation of cracks and destruction of conductive channels. The aforementioned studies had difficulty meeting the requirements of both high sensitivity and wide working range. Therefore, although the sensitivity of the strain sensors based on the crack structure is acceptable, the working range is still unsatisfactory. Nevertheless, we believe that this method is promising as long as the crack morphology can be controlled.

In this article, a flexible strain sensor with ultrahigh sensitivity and a wide working range was designed by combining a stretchable network of electrospun fibers and a microcrack structure. The CNT@AgNW functional layer was loaded on the electrospun TPU fiber mat by vacuum-assisted filtration, and the microcrack structure was constructed by prestretching. The conductive network reinforced by CNT and AgNW entanglements limits slippage so that the loaded force is mainly released by microcrack formation. In addition, CNT&AgNWs form a good interface with the TPU mat, restricting out-of-plane bending and distortion to generate new cracks and expand in new regions. Hence, the working range of this sensor is not limited by the elastic tensile limit of the composite mat; instead, it is controlled by the sum of the width of all the expanded cracks, which is far over the elastic limit. In addition to the wide working range, the sensor showed high sensitivity due to the distributed microcracks. When the sensor is under strain, the number of microcracks increases with load, and the total conductive path forms an “island-bridge” structure. For each crack, the “bridge” at both ends becomes narrower, and the conductive area in the “island” is larger, causing a significant resistance change. Meanwhile, with an increase in the strain load, the conductive paths formed by “island” and “bridge” decrease and further cause resistance variation. Accordingly, a large resistance variation is obtained under strain, resulting in high GF (sensitivity). For comparison, the sensitivity of conventional flexible strain sensors obtained by filling functional nanomaterials in polymeric substrates is limited by the slippage of fillers and the tunneling effect. Finally, the detection capability of this strain sensor for small signals and human joint motion was demonstrated, which provides a feasible path for practical applications in wearable devices to detect human motion.

## Results

### Device fabrication

**Fig. 1:**
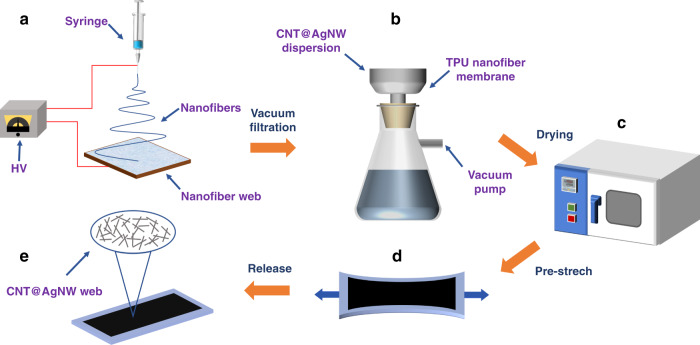
Scheme of the fabrication process of the CNT@AgNW/TPU strain sensor. **a** Preparation of TPU nanofiber mat by electrospinning. **b** Loading of CNT@AgNW layer on TPU nanofiber mat by vacuum-assisted filtration. **c** Drying the composite nanofiber mat. **d** Prestretching the composite nanofiber mat. **e** A flexible strain sensor with microcrack structure was fabricated

The CNT@AgNW/TPU strain sensor was fabricated following the process shown in Fig. 1. TPU porous fiber mats were prepared by electrospinning. First, TPU particles were dissolved in a mixed solution of DMF/acetone (the mass ratio of DMF to acetone was 1:1) with a ratio of 30% wt. The mixture was mechanically stirred for 12 h to obtain a homogeneous spinning solution. Then, the spinning solution was loaded into a 1 ml syringe fixed to a microinjection pump. During the process, the ambient temperature was 20–25 °C, the applied DC voltage was 10 kV, the injection speed of the spinning solution was 1 ml/h, and the distance between the needle tip and the collector was 15 cm. A homemade collecting plate was used to receive fibers, and the prepared fiber mat was air-dried for 24 h.

A small amount of multiwall carbon nanotube (MWCNT) dispersion and AgNW dispersion was dropped into deionized water for ultrasonic treatment for 2 h. Next, 4 mg MWCNT and 2 mg AgNW were deposited on the TPU nanofiber mat by vacuum-assisted filtration. The composite nanofiber mat was placed in an oven and dried at 120 °C for 30 min.

To form a uniformly distributed crack microstructure, the composite mat was slowly stretched to 100% strain at a speed of 4 mm/min by an electronic universal tensile testing machine (PT-1198GDO) and then released to the initial state at the same speed. Crack growth can be controlled by adjusting the prestretching speed and strain value. Then, copper electrodes were connected to the prestretched mat with silver paste to obtain the stable output. The distance between the two electrodes was 30 mm. The prepared CNT@AgNW/TPU strain sensor was labeled CATSS.

### Characterization and discussion

**Fig. 2:**
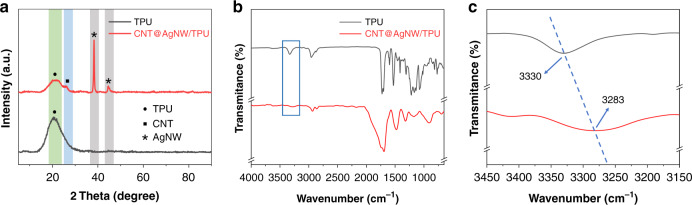
Characterization of the CATSS. **a** XRD; **b** FTIR spectra of the TPU mat before and after loading CNT@AgNW; and **c** an enlarged view of the marked box

The X-ray diffraction patterns of the fabricated samples are shown in Fig. 2a. The characteristic peaks of MWCNTs and AgNWs can be found in the figure (the characteristic peak of MWCNTs appeared at 26.1°, and the peaks of AgNWs appeared at 38.3° and 44.5°), indicating that they were successfully loaded on the TPU mat. For the composite mat prepared as a strain sensor, the interfacial bonding between the conductive layer and the polymer substrate plays a vital role in sensing performance. Figure 2b shows the FTIR spectra of the mat before and after loading CNT@AgNW. 3330 cm^−1^ is the peak of the N-H stretching vibration, as found in the figure. After the deposition of CNT@AgNW, this characteristic peak moves to a lower wavenumber (blueshift, Fig. 2c). The blueshift was attributed to the hydrogen bonds formed between polyvinyl pyrrolidone (PVP) on the surface of AgNW and TPU. The formation of hydrogen bonds by the oxygen-containing groups in the dispersion of MWCNTs with TPU also contributed to the blueshift. Since it is difficult to form chemical bonds between TPU and CNT@AgNW, hydrogen bonding is considered to be a reliable connection between functional materials and elastic substrates.

**Fig. 3:**
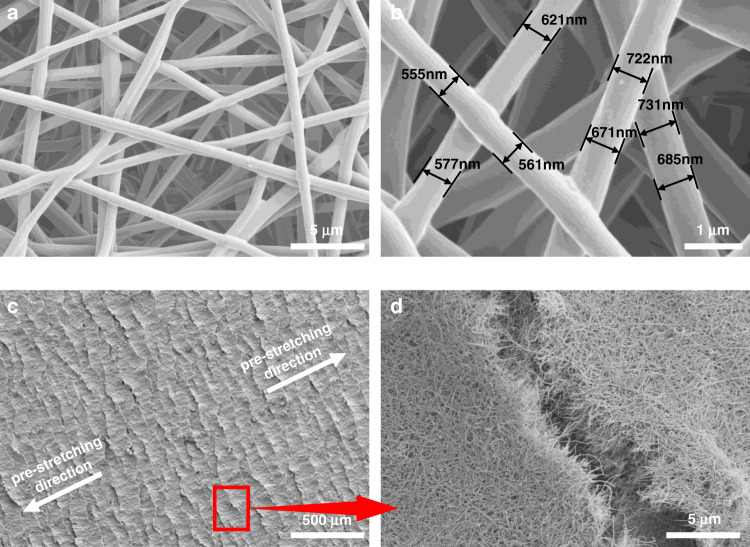
SEM images of the CNT@AgNW/TPU mat. **a**, **b** Porous structure of the electrospun TPU mat. **c** Top view of the prestretched CATSS. **d** Top view of a typical prestretching crack

The morphology of the CNT@AgNW/TPU fiber composite mat was studied, which is of great significance to study the sensing performance. Figure 3a shows the TPU nanofibers prepared by electrospinning. The TPU fibers overlapped with each other and stacked to form a porous structure. Figure 3b shows that the surface of a single fiber is smooth without a bead-on-string structure, which is important to improve the tensile strength. Simultaneously, the diameter of the fibers is mostly distributed between 500 and 750 nm, and such a diameter distribution guarantees the excellent tensile property of the strain sensor. After prestretching the CNT@AgNW/TPU composite mat, obvious microcracks are observed in Fig. 3c with an enlarged view in Fig. 3d. The extension direction of the microcrack was perpendicular to the prestretching direction. The generation of microcracks is indispensable for forming an “island-bridge” structure on the mat, which is the key to obtaining high strain sensitivity.

**Fig. 4:**
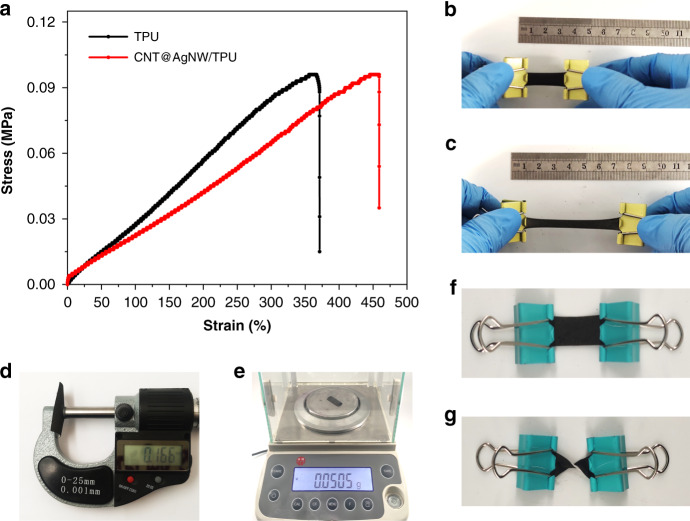
Tensile property and characteristic of the CNT@AgNW/TPU mats. **a** Stress–strain curve (with neat TPU as the control sample); **b** original state; **c** stretched state; **d** ultrathin; **e** ultralight; **f**, **g** ultraflexible

CATSS has excellent stretchability and flexibility. Tensile tests were carried out on neat TPU mats and CNT@AgNW/TPU composite mats. The stress–strain curve obtained at a tensile rate of 5 mm/min is illustrated in Fig. 4a. Compared with the neat TPU fiber mat, the tensile strength of the CNT@AgNW/TPU mat was close, but the strain at break increased obviously, indicating that the carbon nanotubes entering the porous TPU mat during filtration effectively improved the toughness of the composite material. The composite mat can be stretched to over 450% strain, showing exceptional tensile properties. Figure 4b, c shows the tensile situation of the CNT@AgNW/TPU mat. When the mat was stretched to more than 250% strain, there were still no obvious defects on the surface of the mat, providing an extensive strain response range for CATSS. In addition, the composite mat characteristics of being ultrathin, being ultralight, and having excellent flexibility are presented in Fig. 4d–g, paving the way for the practical application of CATSS in tensile strain sensing and wearable flexible electronic devices.

### Sensing performance

**Fig. 5:**
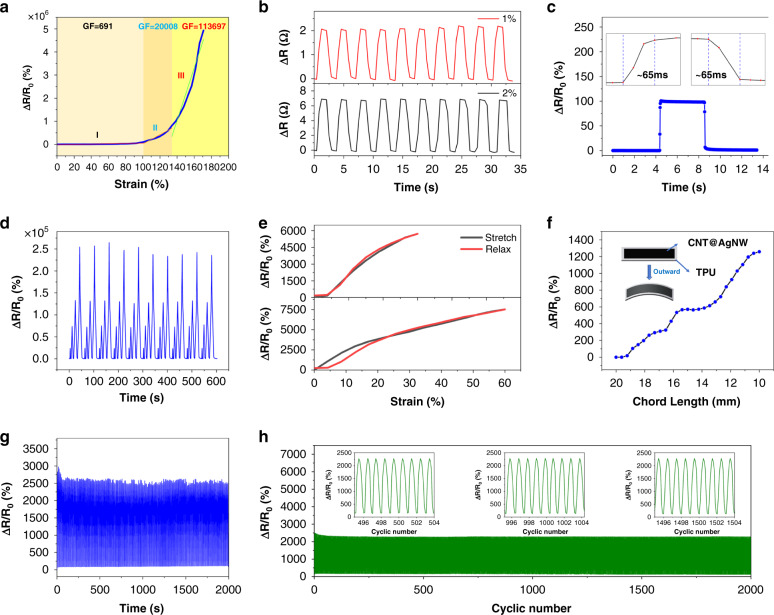
Strain-sensing performance of the CATSS. **a** Δ*R/R*_0_ upon the applied strains for CATSS after 171% strain stretching. **b** Response of CATSS to cyclic loadings at small strains (1 and 2%). **c** Time response of CATSS. Insets: close up of the selected areas. **d** 10-Step strain cycles of strains at 25, 50, 75, and 100%. **e** Hysteresis of CATSS under different strains. **f** Bending strain response of CATSS. **g** Δ*R/R*_0_ of CATSS to cyclic bending loading over 400 cycles. **h** Long-term stability test of a typical CATSS for 2000 stretching-releasing cycles toward the strain variation from 0 to 20%. Insets: close up of the selected areas

Here, the strain-sensing performance of CATSS, including the mechanical-electrical response under tensile and bending strains and the stability in cyclic tests, has been described individually. One key parameter to evaluate sensing performance is the resistance of the composite mat, defined as *R*, while the resistance variation is *ΔR*. As illustrated in Fig. 5a, the curve is divided into three different regions (region I from 0 to 102%, region II from 102 to 135%, and region III from 135 to 171%) and keeps monotonically increasing in each region. Obviously, the sensor achieves a 0~171% sensing range, which is outstanding in the reported work. As mentioned above, the excellent working range of the sensor is attributed to the excellent elongation at break of the neat electrospun TPU mat with a porous network structure. Meanwhile, the ultrahigh sensitivity of CATSS is shown in Fig. 5a. Generally, the sensitivity of the flexible strain sensor is evaluated by GF, which is defined as the ratio of the relative change rate of resistance to the strain of the sensor [GF = ((*R−R*_0_*)/R*_0_*)*/ε], where *R* is the resistance at tension, *R*_0_ is the initial resistance and *ε* is the applied strain. The strain sensor possesses superb sensitivity with a GF of 691 in region I, ~2 × 10^4^ in region II, and >11 × 10^4^ in region III. The rapid increase in GF is attributed to the crack propagation caused by the increase in strain. With crack evolution, the number of conductive paths decreased sharply, which increased the resistance and eventually led to a rapid increase in GF.

Tests were performed on CATSS to systematically interpret the performance of the sensor. To verify the high sensitivity of the sensor, the detection ability of the sensor to small strain is reflected in Fig. 5b, and an obvious change in the resistance can be observed even under 1% strain. Moreover, regardless of whether 1 or 2% strain was applied, the signal waveform was basically maintained. We keep the strain for 1 s after each stretch, and a fairly flat tip can be observed, which suggests that the sensor has a stable recognition ability for small deformations. At the same time, in Fig. 5c, the sensor withstood a 1% quasi-transient step strain, and the response and recovery time of the sensor were ~65 ms, which are superior to the existing reports. Furthermore, the speedy response of CATSS is of great significance for its real-time monitoring of human motion behavior. In addition, through the test of the single tensile-recovery cycle of CATSS, it can be seen (Fig. 5d) that the curves representing the tensile and recovery almost coincide, indicating that the hysteresis of the sensor is very small. To further validate the good detection of CATSS to large strain behavior, cyclic strains in gradually increasing steps were applied, as shown in Fig. 5d, and the response of the sensor in cycles at large strains of 25, 50, 75, and 100% is shown. Excellent consistency of the signals was observed across multiple cycles, indicating that the sensor had good reliability for large strain detection.

In addition, to make the sensor suitable for more application scenarios, we explored the output performance of the sensor under bending. One sensor was pasted onto a thin polyimide mat, and copper electrodes were connected to the mat using conductive silver glue. During the bending strain test, the sensitivity of the sensor is also defined as the ratio of the relative change of the resistance to the strain [GF = ((*R−R*_0_)/*R*_0_*/ε*_*l*_)], where the definition of *R* and *R*_0_ is similar to the tensile test. In particular, the strain here refers to the change in the chord length; in other words, the strain can be calculated by the following formula: *ε*_*l*_ = *(l−l*_0_*)/l*_0_. Then, the relationship between the chord length *l* and the curvature radius of the mat during bending is *l* = *2r*sin*(L/*2*r)*, where *L* refers to the length of the sensor strip. When the sensor strip is bent outward, the sensor exhibits a similar linear monotonically increasing curve in Fig. 5f, which should also be attributed to the propagation of the sensor microcracks. When the sensor is bent outward, although the total length of the strip sensor is unchanged, due to the large modulus mismatch between the TPU mat and the CNT@AgNW composite layer, the prefabricated crack will continue to expand, resulting in a decrease in the conductive path and then causing a rapid increase in resistance. In addition, the bending-recovery cycle test of the sensor was carried out. The results in Fig. 5g show that the bending sensing behavior of the sensor is stable and repeatable, indicating that the sensor has a good capability to detect bending deformation.

Finally, maintaining long-term reliable operation of the strain sensor in practical application is of significance. Therefore, the cyclic stability of CATSS was tested to avoid performance degradation caused by the long-term operation of the sensor. To investigate the long-term stability of CATSS, a periodic stretching-releasing test with 2000 cycles was performed under 20% strain at a speed of 2 mm/s. The curve in Fig. 5e shows that in 2000 cycles, there is no obvious signal fluctuation, indicating that the sensor has good durability and reliability.

### Sensing mechanism

#### Wide working range

**Fig. 6:**
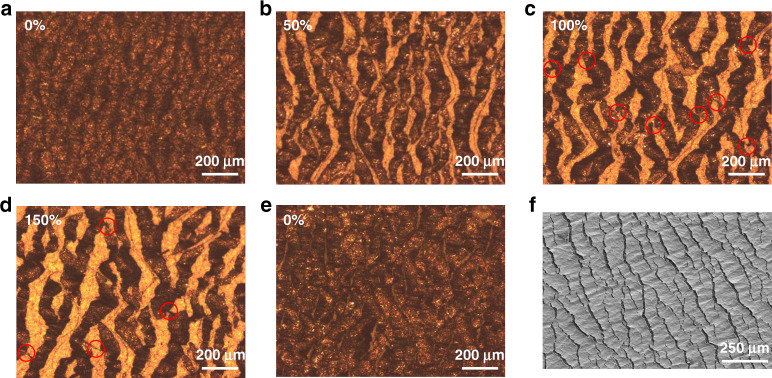
Optical images of crack distribution under different strains. **a** Initial; **b** 50% strain; **c** 100% strain; **d** 150% strain; and **e** recovery to the initial state. **f** SEM of CATSS under 50% stretching strain

An island-bridge structure was formed in the conductive layer. Specifically, the formation of microcracks divides the CNT@AgNW conductive layer into isolated islands, and the conductive materials at both ends of the crack and the part extending from the edge of the crack serve as bridges to connect the islands. The evolution of the microcrack structure was studied by an optical microscope, and the results are illustrated in Fig. 6. Figure 6a shows an image of the prestretched mat. Then, the prestretched mat was stretched to 50, 100, and 150% strain. Figure 6b–d, f shows the crack propagation in each state. Apparently, the crack area expands with increasing strain. Specifically, the crack width increases along the tensile direction, and the crack length gradually increases in the direction perpendicular to the tensile direction, leading to crack structure expansion.

With good interface bonding, the conductive layer seldom experiences out-of-plane bending or torsion, and crack propagation is constrained in the plane. In the initial stage, when the strain increases, the large strain is transformed into a small deformation of each crack by prestretching. Although there is stress concentration at the slit on both ends of the crack, the crack propagation speed is slow because the deformation of a single crack is small. No long crack that can destroy the whole conductive path was formed. When the strain increases to a certain extent, the in-plane deformation will be unstable due to the difference in material modulus, and the conductive network has the trend of out-of-plane bending or torsion^42^. However, the good interface bonding between the substrate and the conductive network will inhibit this trend and make new cracks produce and expand in new regions^43^. This ensures the integrity of the conductive network under large strain. When the deformation is recovered, the cracks are almost restored to the initial shape, and most of the conductive paths are restored. The sensor output will recover to the initial value. This is one major reason for the wide working range of this strain sensor.

### High sensitivity

**Fig. 7:**
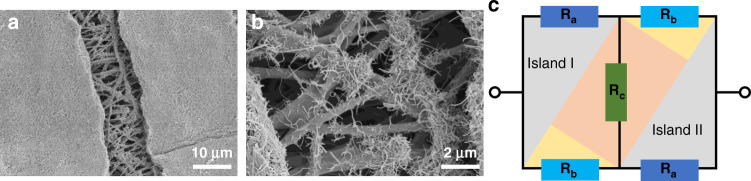
The strain-sensing mechanism of the CATSS. **a**, **b** Micromorphology of a typical “island bridge” structure; **c** resistance model of the “island bridge” structure

Combined with a previous investigation on the crack propagation behavior of the sensor, a typical microcrack is selected in the tensile process, as shown in Fig. 7a, to analyze the sensing mechanism. In Fig. 7a, an ‘island bridge’ microstructure can be clearly observed. Undoubtedly, two islands are connected by bridge-shaped CNT@AgNW at each end of the crack. Simultaneously, CNT@AgNW loaded on TPU fibers can connect islands, and their existence can be intuitively observed in Fig. 7b. To clearly explain this mechanism, we use the model developed by Yamada et al. to simplify the resistance change of the crack structure into the following expression^44^, where *R*_*a*_ represents the resistance of the island, *R*_*b*_ represents the resistance of the bridge connecting the two islands at both ends of the crack, and *R*_*c*_ represents the resistance of CNT@AgNW loaded on a TPU fiber. *R’* is the resistance of a single microcrack structure.

1 $$R^\prime = \frac{{R_aR_c + 2R_aR_b + R_bR_c}}{{R_a + 2R_c + R_b}}$$

It should be noted that after the prestrain release, there are a large number of overlapping parts of CNT@AgNW between the small overlap (wrinkles) on the sample surface. During the tensile process, the overlapping area of adjacent CNTs and AgNWs changes, which leads to a change in the contact resistance. Compared with the resistance change caused by the crack structure, that caused by the slip of CNTs or AgNWs can be neglected. When the strain increases, based on the above crack propagation mechanism, the connection part between the two islands becomes narrower, and the TPU fiber loaded with conductive materials becomes longer, which contributes to the simultaneous increase in *R*_*b*_ and *R*_*c*_. As the crack propagates in the direction perpendicular to the pretension, the width of the bridge will inevitably decrease, resulting in a reduction in the conductive path and an increase in *R*_*b*_. It can be considered that *R*_*b*_ is positively correlated with strain. At the same time, with increasing strain, TPU fibers loaded with CNT@AgNW become longer, hindering electron transfer. Accordingly, *R*_*c*_ can also be considered positively correlated with strain. Moreover, the change in *R’* should follow percolation theory^45^. Therefore, the resistance *R’* should also show the changing trend of a quasi-exponential function^44^, which provides a high GF for the sensor. This analysis is consistent with the measurement results in Fig. 5a. Ultimately, the flexible strain sensor in this work combines ultrahigh sensitivity with a wide strain working range through a microcrack structure design.

### Application

**Fig. 8:**
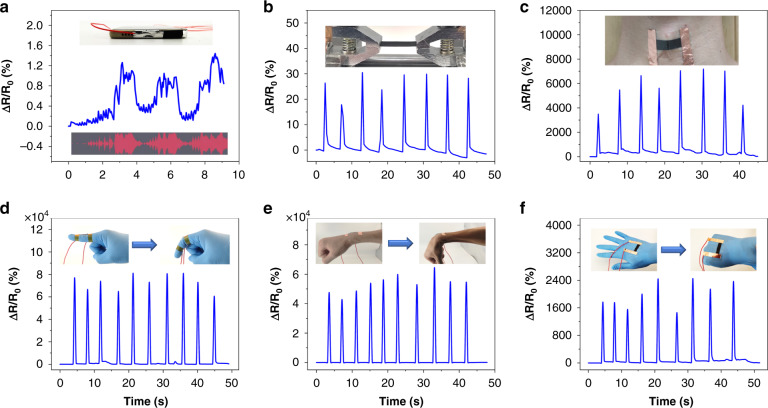
Sensing applications of the CATSS. **a** Recognition of sound signals from mobile phone loudspeakers. **b** Recognition of weak airflow impact from the ear washing ball. **c** Recognition of volunteers swallowing food. **d** Recognition of finger bending. **e** Recognition of wrist bending. **f** Changes in the Δ*R/R*_0_ of the sensor attached to the back of the hand when the volunteer clenched his fists

For CATSS, the ultrahigh sensitivity and wide working range enabled it to detect various motion signals. The sensor was pasted on the loudspeaker of a smartphone, and the vibration of the sensor was driven by the sound signal emitted by the smartphone. Figure 8a shows that the sensor has an instant response to the vibration driven by the sound signal. When the phone played audio, the collected signal was synchronized with the original signal. Then, the sensor was fixed in the horizontal direction through a fixture, and airflow impact was produced by squeezing an ear washing ball. Figure 8b shows the sensitive response of the sensor to airflow impact, indicating that CATSS has a high resolution in motion monitoring.

Furthermore, CATSS has broad application prospects in human motion detection. As shown in Fig. 8c, when the sensor was connected to a volunteer’s throat, the swallowing behavior of food or water could be detected. CATSS also had a good response for human motion with large deformation. For example, by pasting the sensor onto the finger and wrist, as shown in Fig. 8d and e, the sensor can continuously monitor movement of the finger and the wrist when volunteers bent their finger or wrist. Finally, the sensor was pasted on the back of a hand, and the action from loosening the hand to shaking the fist could be captured. All the above results show that CATSS can be used to detect small motion signals or large-scale human motion, which confirms that the designed flexible strain sensor based on the microcrack structure has diverse potential applications in wearable electronics, human motion monitoring, and beyond.

## Conclusion

A flexible strain sensor based on the microcrack structure was fabricated through a simple and efficient strategy: vacuum-assisted filtering and prestretching technology. The sensor has high sensitivity (GF of 691 within 0~102% strain, ~2 × 10^4^ within 102~135% strain, and >11 × 10^4^ within 135~171% strain), a wide working range (0~171% strain), and excellent cyclic stability (>2000 cycles). The good sensing performance is attributed to the outstanding mechanical properties of the electrospun fiber mat and the microcrack propagation mechanism based on the “island-bridge” model. In addition, the application of the sensor in weak signal capture and large-scale human motion monitoring is demonstrated, showing the brilliant prospects of flexible strain sensors in wearable electronic products.

## Methods

### Materials

A carbon nanotube aqueous dispersion (MWCNTs, diameter = 30 nm, length = 100 μm, 10 wt%) was purchased from XFNANO (Nanjing, China). Ag nanowire aqueous dispersion (AgNW, diameter = 30 nm, length = 30 μm, 10 mg/ml) was purchased from Puwei Nano Technology (Shanghai, China). Thermoplastic polyurethane (TPU, code Elastollan 1185 A) provided by BASF Co. Ltd. N,N-dimethylformamide (DMF, 99%) was purchased from Aikang Biomedical R&D Co., Ltd. (Nanjing, China). Acetone was ordered from Aladdin Biochemical Technology Co., Ltd. (Shanghai, China).

### General characterization techniques

The surface morphology of the samples was analyzed using field-emission scanning electron microscopy (FE-SEM, SU-8010) at a 5 kV accelerating voltage. X-ray diffraction patterns of the samples were obtained by an X-ray diffractometer (D8 ADVANCE A25). The 2θ angle was scanned in the range of 5~90°. Fourier transform infrared (FTIR) spectra of the samples were recorded on an FTIR spectrometer (Nicolet iS50, ranging from 650 to 4000 cm^−1^ with a resolution of 4 cm^−1^). Stress–strain curves of the material were tested by a universal tensile testing machine (PT-1198GDO). The mechanical and electrical properties of the strain sensor were measured using self-built material tensile equipment and a multifunctional digital multimeter (Keithley DAQ6510 and Keysight 34460 A). The evolution of the crack structure during the deformation process was recorded by an optical microscope.

## Supplementary information

Supplemental figures and tables
