# Supplementary material for: An ultrasensitive and stretchable strain sensor based on a microcrack structure for motion monitoring
Source: Microsyst Nanoeng. 2022 Sep 29;8:111. doi: 10.1038/s41378-022-00419-6 (PMC9522852; doi:10.1038/s41378-022-00419-6)
Supplement: Supplementary file 1 — Supplemental figures and tables [file 41378_2022_419_MOESM1_ESM.docx]

***Supporting Information***

**An ultra-sensitive and stretchable strain sensor based on micro-crack structure for motion monitoring**

Hao Sun^1,2#^, Xudong Fang^1,2#^, Ziyan Fang^1,2^, Libo Zhao^1,2,3^, Bian Tian^1,2,3^, Prateek Verma^4^, Ryutaro Maeda^1,2^ and Zhuangde Jiang^1,2^

*^1^State Key Laboratory for Manufacturing Systems Engineering, International Joint Laboratory for Micro/Nano Manufacturing and Measurement Technologies, Collaborative Innovation Center of Suzhou Nano Science and Technology, Xi’an Jiaotong University, Xi’an 710049, China*

*^2^School of Mechanical Engineering, Xi’an Jiaotong University, Xi’an 710049, China*

*^3^Overseas Expertise Introduction Center for Micro/Nano Manufacturing and Nano Measurement Technologies Discipline Innovation, and Xi’an Jiaotong University (Yantai) Research Institute for Intelligent Sensing Technology and System*

*^4^School of Chemical Engineering, University of Arkansas, AR 72701, USA*

*^#^These authors contributed equally: Hao Sun, Xudong Fang.*

*^*^E-mail: dongfangshuo30@xjtu.edu.cn*


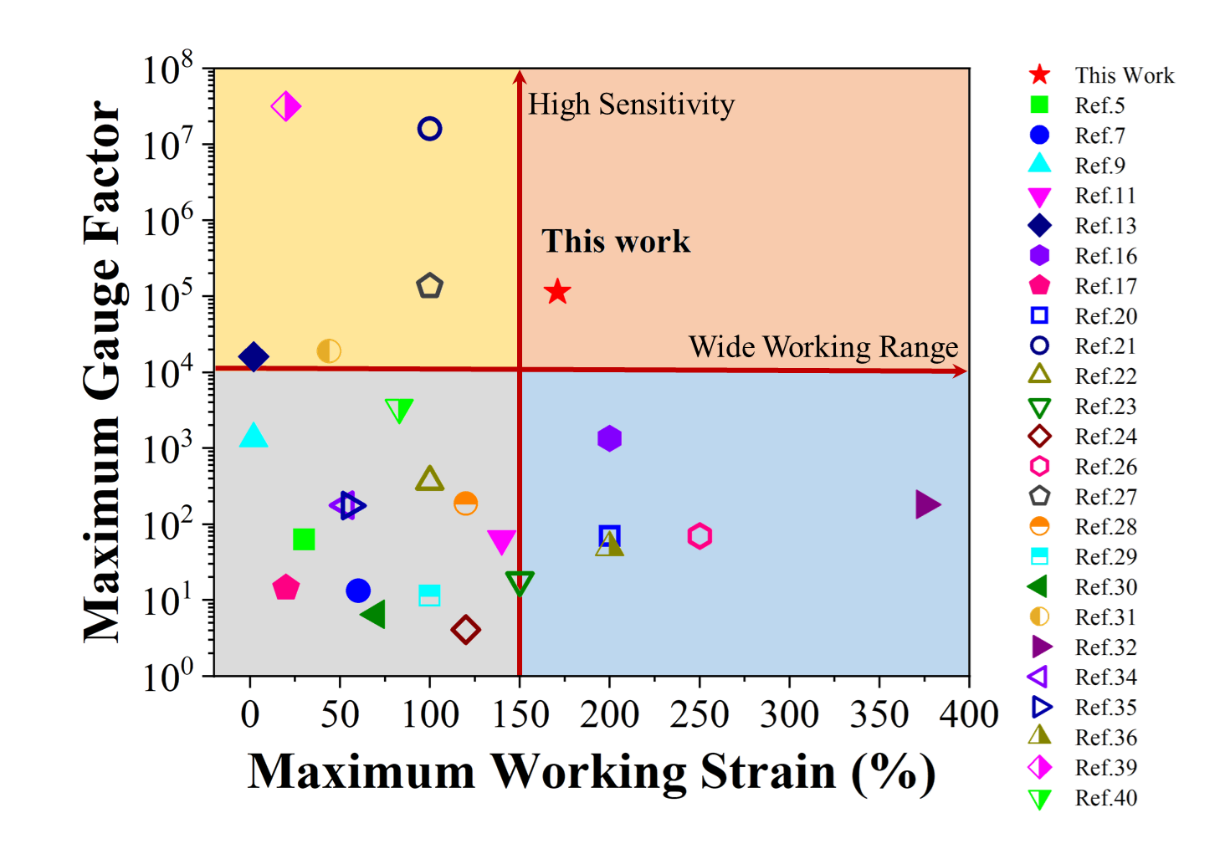


Figure S1. Comparison of sensitivity and working range of sensors

Figure S1 shows the comparison of sensitivity and working range between the sensor in this work and related flexible strain sensors in recently published papers. The GF of 5 other sensors exceeds 10^4^, and 6 other sensors achieve a wide working range of more than 150% strain. However, only our sensor has the high sensitivity over 11×10^4^ under the maximum working range of 171%, achieving high sensitivity and wide working range at the same time. By comparing the related work in recent years, our strategy is confirmed effective in resolving the conflict between high sensitivity and wide working range of flexible strain sensors.

Table S1. Summary of main strain sensing properties of recently reported flexible strain sensors.

| **Materials** | **Gauge Factor** | **Maximum Working Range** | **Response Time** | **Cyclic Number** | **Reference** |
| --- | --- | --- | --- | --- | --- |
| **CNT/AgNW/TPU** | **1.1×10^5^** | **171%** | **65ms** | **2000,20%** | **This Work** |
| CB/spandex/polyamide fabric | 62.9 | 30% | / | 20000,10% | 5 |
| CNT/Ti_3_C_2_T_x_ MXene/PDMS | 13.3 | 60.30% | / | 1000,30% | 7 |
| CNT/PLA | 1342.1 | 2% | / | 2300,2% | 9 |
| Carbonized plain weave cotton fabric | 64 | 140% | / | 2000,50% | 11 |
| Pt/PUA/PET | 16000 | 2% | / | / | 13 |
| CNT/PU yarn | 1344.1 | 200% | 88ms | 10000,30% | 16 |
| CNT/Ecoflex | 14.5 | 20% | / | 50,25% | 17 |
| Graphene/Silk fibroin | / | 100% | 7ms | 1000,50% | 19 |
| AgNW/Agel | 70 | 200% | / | 500,60% | 20 |
| AgNW/MXene/WPU | 1.6×10^7^ | 100% | 344ms | 1000,8% | 21 |
| CNT/MXene/TPU | 363 | 100% | / | 200,80% | 22 |
| Mxene/Polyacrylamide-sodium hydrogel | 18.15 | 150% | 74ms | / | 23 |
| Mxene/AgNPs/P(AAm-co-HEMA) hydrogel | 4.08 | 120% | 120ms | 400,50% | 24 |
| AgNW/PU | 70 | 250% | / | 500,20% | 26 |
| CNT/AgNW/TPU/PDMS | 1.36×10^5^ | 100% | 181ms | 1200,70% | 27 |
| CNT/Graphene/PDMS | 186.5 | 120% | 60ms | 10000,40% | 28 |
| AgNW/PET/PDMS | 11.4 | 100% | / | 1700,30% | 29 |
| CNF/PDMS | 6.5 | 70% | 160ms | 10000,30% | 30 |
| Graphene/CB/Silicone rubber | 1.89×10^4^ | 44% | / | 1000,5% | 31 |
| CF/CB/Silicone rubber | 182 | 375% | / | 200160% | 32 |
| Ti3C2Tx MXene/PDMS | 178.4 | 53% | / | 5000,20% | 34 |
| Braided graphene belts/Dragon skin | 175.16 | 55.55% | 120ms | 6000,30% | 35 |
| Cu/PVA | 49.5 | 200% | 60ms | 3000,50% | 36 |
| AgNW/Acrylate | 3.2×10^7^ | 20% | 14.4ms | / | 39 |
| MXene/Cellulose nanocrystal/TPU | 3405 | 83% | 75ms | 1000,10% | 40 |
